# Incomplete posttranslational prohormone modifications in hyperactive neuroendocrine cells

**DOI:** 10.1186/1471-2121-10-35

**Published:** 2009-05-07

**Authors:** Jeroen RPM Strating, Gerard JM Martens

**Affiliations:** 1 Department of Molecular Animal Physiology, Donders Institute for Brain, Cognition and Behaviour, and Nijmegen Centre for Molecular Life Sciences (NCMLS), Radboud University Nijmegen, RT 282, Geert Grooteplein Zuid 28, 6525 GA Nijmegen, The Netherlands; 2 Current address: Heidelberg University Biochemistry Center (BZH), Im Neuenheimer Feld 328 - #224, D-69120 Heidelberg, Germany

## Abstract

**Background:**

In black-background-adapted *Xenopus laevis*, the intermediate pituitary melanotrope cells are hyperactive, producing large amounts of their major secretory cargo proopiomelanocortin (POMC, representing ~80% of all newly synthesised proteins), whereas in white-adapted frogs these cells are only basally active. Here we explored in the hyperactive and basally active melanotrope cells the capacity for posttranslational POMC processing events in the secretory pathway.

**Results:**

We found that the hyperactive cells produced mainly non-complex N-glycosylated POMC, whereas in the basally active cells POMC was mostly complex N-glycosylated. Furthermore, the relative level of POMC sulphation was ~5.5-fold lower in the hyperactive than in the basally active cells. When the cargo load in the secretory pathway of the hyperactive cells was pharmacologically reduced, the relative amount of complex glycosylated POMC markedly increased.

**Conclusion:**

Collectively, our data show that the secretory pathway in hyperactive neuroendocrine secretory cells lacks the capacity to fully comply with the high demands for complex glycosylation and sulphation of the overload of secretory cargo. Thus, a hyperactive secretory cell may run short in providing an output of correctly modified biological signals.

## Background

A variety of vertebrate secretory cells with a basal activity become highly activated during their development or maturation, such as the immunoglobulin-secreting plasma cells and the insulin-secreting pancreatic beta-cells. Induction of cells to become professional secretors involves massive changes at the level of gene transcription, protein biosynthesis and cellular ultrastructure. Secretory organelles such as the endoplasmic reticulum, the Golgi apparatus and the secretory granules become expanded and a variety of genes is upregulated, including those encoding the secretory cargo and the machinery involved in the transport and biosynthesis of the cargo (see for example [1,2]). We use a unique neuroendocrine cell model, namely the *Xenopus laevis *intermediate pituitary melanotrope cells, to study the events occurring during the activation of neuroendocrine cells to become professional secretory cells. The melanotropes are inducible neuroendocrine cells that play a central role in the process of background adaptation of the animal. When the frogs are on a white background (white adapted; WA), the melanotrope cells are only basally active. Placing the animals on a black background (black adapted; BA) physiologically activates the melanotrope cells to become hyperactive professional secretory cells. Hyperactive melanotropes are dedicated to produce and proteolytically process vast amounts of the prohormone proopiomelanocortin (POMC) and secrete the POMC-derived products (reviewed in [3]). In *Xenopus *melanotrope cells, POMC is synthesised as an N-glycosylated and sulphated 37 K prohormone [4,5] that is subsequently cleaved to generate a number of bioactive peptides. The C-terminal portion of POMC is processed to α-melanophore-stimulating hormone (MSH), β-MSH, corticotropin-like intermediate lobe peptide (CLIP), β-endorphin and some linking regions of unknown function [4,6,7]. The N-terminal part of POMC (18 K POMC), which bears the only N-glycan present in *Xenopus *POMC, can be cleaved to two different γ-MSH peptides, although the majority (90–95%) is not processed to γ-MSH but secreted as glycosylated 9 K or 18 K products [8]. MSHs released into the blood cause darkening of the skin to allow the animal to adapt to its background.

When considering neuroendocrine cell activation one wonders whether basally active secretory cells that are physiologically activated do not only change the quantity but also the quality of their secretory output. A change in output quality would imply an involvement of the machinery responsible for the biosynthesis of the secretory cargo. In this study we tested this hypothesis by exploring the biosynthetic machinery in the basally active and hyperactive *Xenopus *melanotrope cells, in particular the implications of secretory cell induction of the Golgi-based posttranslational processing of secretory cargo proteins. We find profound differences in POMC N-glycosylation and sulphation between the hyperactive and the basally active melanotrope cells. Our experiments indicate that in the hyperactive melanotropes the Golgi apparatus lacks sufficient glycosylation and sulphation capacity to properly process the high amounts of newly synthesised POMC.

## Results

### Biosynthesis of 18 K and 18 K* POMC in the melanotrope cells of black- and white-adapted *Xenopus*

**Figure 1:**
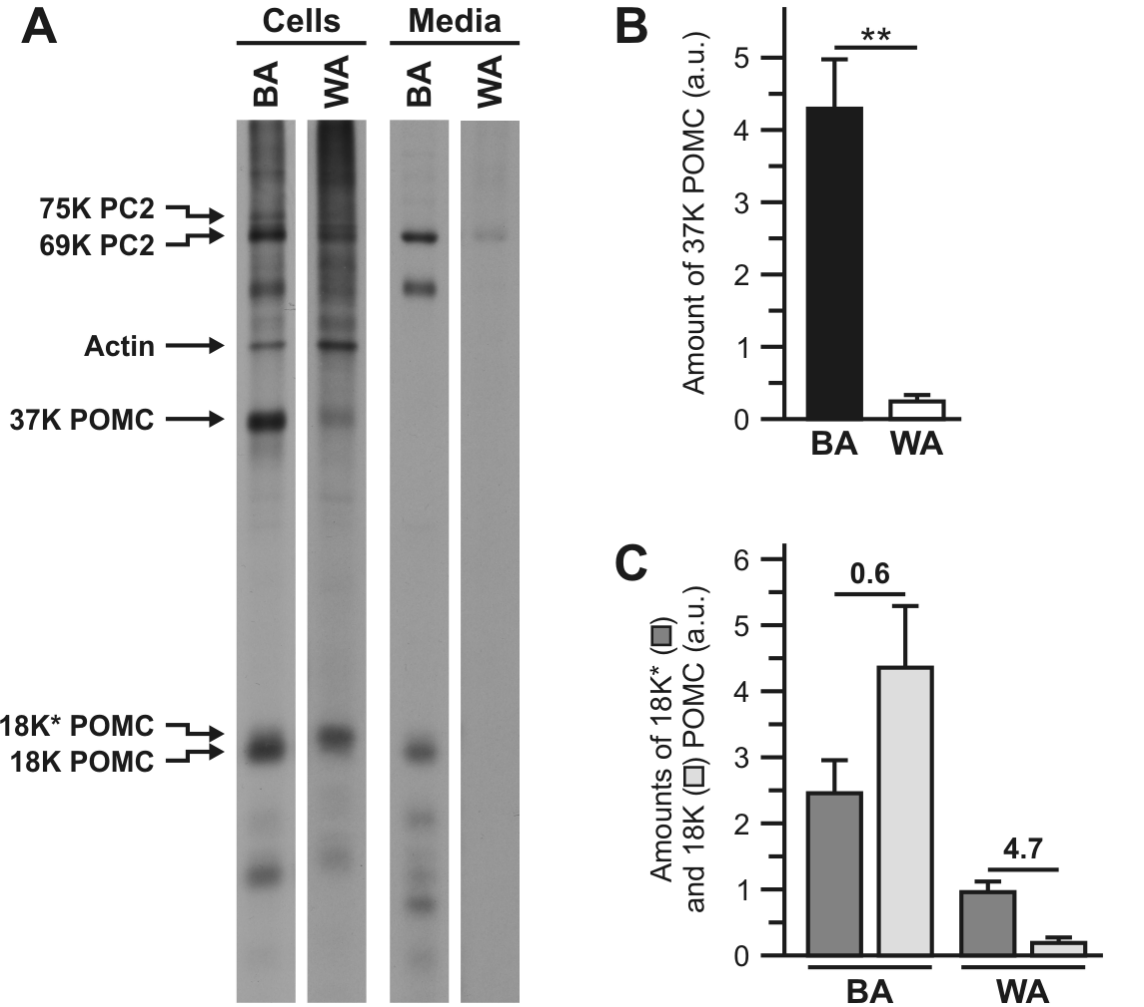
**Biosynthesis of POMC in hyperactive and basally active *Xenopus *melanotrope cells**. **A**, Neurointermediate lobes (NILs) from black-adapted (BA) and white-adapted (WA) animals were pulse labelled with [^35^S]methionine/cysteine for 30 minutes and subsequently chased for three hours. The WA NILs were pulsed and chased in the presence of 10^-6 ^M apomorphine to retain their basally active characteristics. Aliquots of the cell lysates (cells; BA: 5% of total lysate, WA: 10%) and the incubation media (media; BA: 10%, WA: 20%) were analysed by 15% SDS-PAGE and autoradiography. **B**, The amount of 37 K POMC remaining in the cells following the pulse-chase incubation. **C**, The total amounts (cells + media) of 18 K and 18 K* POMC produced during the pulse-chase incubations. The 18 K*/18 K ratios are given above the bars. Data are shown as means +/- s.e.m.; **, p < 0.01; all bars represent four animals.

Previous biosynthetic studies have shown that in *Xenopus *melanotrope cells POMC is initially synthesised as a 37 K prohormone that is subsequently cleaved to an N-terminal, glycosylated ~18 K POMC protein [8]. In activated melanotrope cells, two forms of the ~18 K product exist, namely a major product (~75%) named 18 K POMC and a minor product (~25%) termed 18 K* POMC that has a slightly higher apparent molecular weight [9]. The 18 K and 18 K* POMC products have the same protein backbone and differ only in their N-glycans [10]. To investigate the production of the 18 K and 18 K* POMC products in the hyperactive melanotrope cells from BA frogs (hereafter referred to as BA melanotrope cells) and the basally active melanotrope cells from WA animals (WA melanotrope cells), we used metabolic cell labelling to perform biosynthetic pulse-chase analyses. Following a 30 minutes pulse and three hours chase period, newly synthesised 18 K POMC represented the major ~18 K POMC-derived product in both the BA melanotrope cells and their medium. In contrast, the WA melanotrope cells produced and secreted almost exclusively the 18 K* POMC product (Figures 1A and 1C). Apomorphine did not significantly affect the biosynthesis and processing of POMC, but effectively inhibited secretion from the BA and the WA cells (not shown), indicating that in the BA and the WA cells the POMC-derived products were released via the regulated secretory pathway.

### Dynamics in the biosynthesis of 18 K and 18 K* POMC in the melanotrope cells of black-adapted *Xenopus*

**Figure 2:**
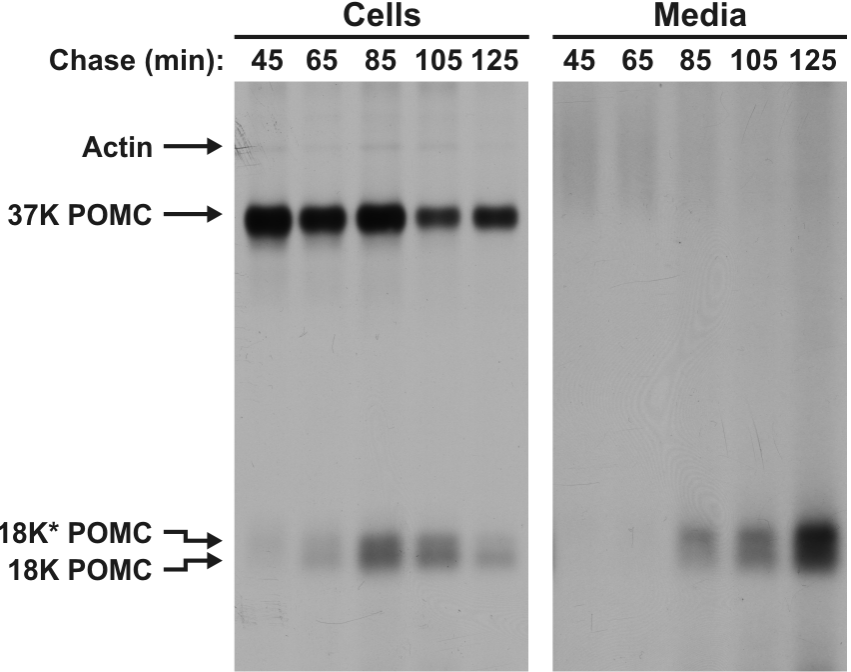
Dynamics of 18 K and 18 K* POMC biosynthesis in hyperactive *Xenopus *melanotrope cells. Neurointermediate lobes from black-adapted *Xenopus laevis *were pulse labelled with [^35^S]methionine/cysteine for ten minutes and chased for the indicated time periods. Aliquots of the cell lysates (10%) and the incubation media (20%) were analysed by 15% SDS-PAGE and autoradiography.

Next, we wondered about the dynamics of 18 K and 18 K* POMC biosynthesis in the hyperactive melanotrope cells. For this purpose, we pulse labelled neurointermediate lobes (NILs) from BA animals for ten minutes and chased the lobes for various time periods. Interestingly, following short chase periods 18 K* POMC was the most prominent product produced in the melanotrope cells, whereas after longer chase periods 18 K POMC became more prominent. 18 K* POMC was initially also the major product released into the medium, but following the longer chase periods 18 K POMC became the prominent product released (Figure 2). Thus, the initial appearance of mainly 18 K* in the hyperactive cells and media shows that the production of 18 K* POMC is faster than that of 18 K POMC. WA melanotrope cells essentially produce only 18 K* POMC and thus no dynamics in 18 K/18 K* POMC biosynthesis was observed in these cells.

### Glycosylation of 18 K and 18 K* POMC in the melanotrope cells of black- and white-adapted *Xenopus*

**Figure 3:**
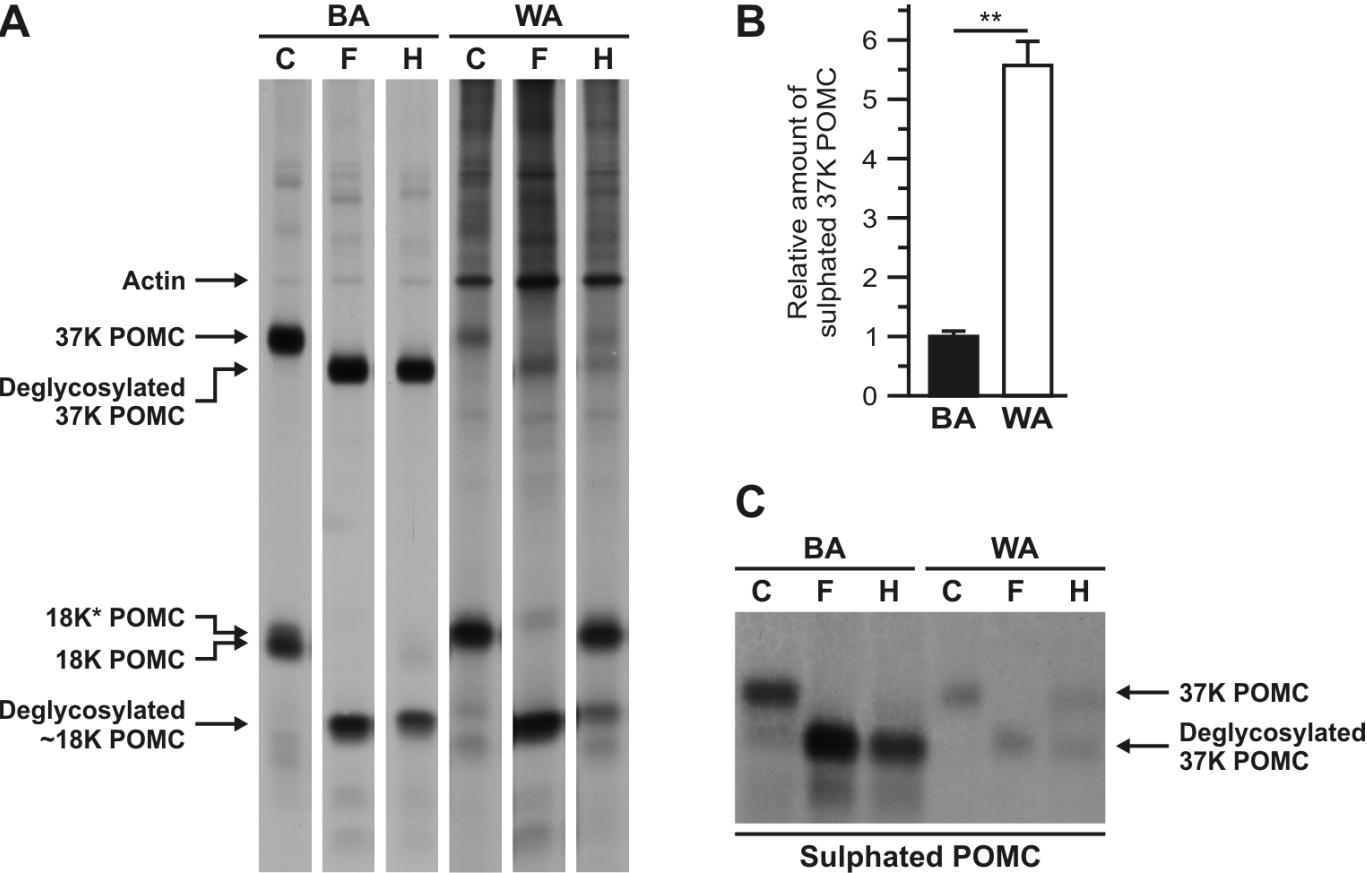
**Glycosylation and sulphation of POMC in hyperactive and basally active *Xenopus *melanotrope cells**. **A**, Neurointermediate lobes (NILs) from black-adapted (BA) and white-adapted (WA) animals were pulse labelled with [^35^S]methionine/cysteine for 60 minutes and chased for two hours. The WA NILs were pulsed and chased in the presence of 10^-6 ^M apomorphine to retain their basally active characteristics. NIL proteins were control treated (C) or deglycosylated with Peptidyl N-glycosidase F (PNGaseF; F) or Endoglycosidase H (EndoH; H), and subsequently analysed by SDS-PAGE and autoradiography. **B**, Newly synthesised proteins in NILs from BA (n = 12) and WA (n = 4) animals were double-labelled with [^3^H]lysine and [^35^S]sulphate for 15 minutes. The WA NIL proteins were labelled in the presence of 10^-6 ^M apomorphine. NIL proteins (40% of the cell lysate) were separated by 12.5% SDS-PAGE and the relative amount of each label incorporated in newly synthesised 37 K POMC was determined. **C**, NIL proteins from BA and WA animals were labelled as in B, control treated (C) or deglycosylated with PNGaseF (F) or EndoH (H), and analysed by 12.5% SDS-PAGE and autoradiography. Data are shown as means +/- s.e.m.; **, p < 0.01.

We then deglycosylated the newly synthesised proteins produced in the BA and the WA melanotrope cells with Peptidyl N-glycosidase F (PNGaseF), an enzyme that removes all N-linked glycogroups irrespective of their composition. We found that in each sample the 18 K and 18 K* POMC products were shifted to a single ~15.5 K product (Figure 3A). Thus, the 18 K and 18 K* POMC products from the BA as well as from the WA cells differ only in their N-linked glycogroups.

To examine the difference in the glycogroups in more detail, we deglycosylated the newly synthesised proteins with Endoglycosidase H (EndoH), an enzyme that selectively removes high-mannose N-glycans. We observed that in BA NILs 37 K POMC was EndoH-sensitive (Figure 3A), suggesting that it had not yet undergone complex glycosylation. Since the proteolytic cleavage of 37 K POMC starts already in the *trans*-Golgi network (TGN) and continues in the TGN-derived immature secretory granules [11], 37 K POMC is proteolytically processed soon after it has encountered the Golgi-enzymes that process the N-glycans to EndoH-resistant conformations. Therefore, the majority of the EndoH-sensitive 37 K POMC molecules may have resided in a pre-Golgi compartment and may not yet have encountered the enzymes that confer EndoH-resistance. To prevent the proteolytic processing of 37 K POMC and thus cause the prohormone to accumulate in a post-Golgi compartment, we performed pulse-chase experiments with BA NILs in the presence of bafilomycin, a drug that inhibits acidification of the TGN/secretory granules. Surprisingly, in the presence of bafilomycin the amount of EndoH-resistant 37 K POMC increased only marginally (not shown). In line with this observation, the vast majority of the 18 K POMC processing product that was produced in the absence of bafilomycin was EndoH-sensitive as well (Figure 3A). These findings suggest that BA melanotropes are unable to convert the N-glycans on POMC to complex glycogroups. Remarkably, in WA melanotropes a significant portion of the 37 K and 18 K* POMC molecules was EndoH-resistant (Figure 3A) showing that, while the prohormone passes through the Golgi apparatus, the WA cells possess the capability to convert a substantial portion of the N-glycans of POMC to a complex form. Interestingly, a part of the 18 K* POMC molecules in WA cells was still EndoH-sensitive. Hence, 18 K* POMC does apparently not represent a single product but rather constitutes 18 K POMC products with slightly different N-glycans. Possibly, these differentially glycosylated 18 K* POMC products represent intermediates of high-mannose to complex glycosylated 18 K POMC products.

### Sulphation of 37 K POMC in the melanotrope cells of black- and white-adapted *Xenopus*

In addition to N-glycosylation, POMC is also posttranslationally modified by sulphation. We therefore wondered whether POMC produced in BA and WA melanotropes differs not only in its glycosylation but also in its sulphation state. To determine the relative amount of POMC-sulphation, we pulse labelled the cells for 15 minutes simultaneously with [^3^H]lysine and [^35^S]sulphate, and determined the amount of each label incorporated into newly synthesised 37 K POMC. We observed that the relative amount of sulphated 37 K POMC was ~5.5-fold higher in WA than in BA cells (Figure 3B). Sulphate-modifications can be attached directly to the protein backbone through a tyrosine residue or indirectly through N-glycans.

Deglycosylation of [^35^S]sulphate labelled 37 K POMC with PNGaseF did not reduce its level (Figure 3C), indicating that *Xenopus *POMC is not glyco- but rather tyrosine-sulphated. The Sulfinator prediction program [12] indeed predicts a sulphation site on tyrosine residue 188 (SLELDY^188^PEIDLDEDIED) in the C-terminal half of *Xenopus *37 K POMC.

Deglycosylation with EndoH revealed that essentially all sulphated POMC from BA cells was EndoH-sensitive, whereas ~50% of the sulphated POMC from WA cells was EndoH-resistant (Figure 3C). The ratio of EndoH-sensitive to -resistant [^35^S]sulphate-labelled 37 K POMC from WA cells is similar to that of [^35^S]methionine/cysteine-labelled 37 K POMC from WA cells (compare Figures 3A and 3C), suggesting that the sulphation machinery does not discriminate between complex and non-complex glycosylated 37 K POMC.

### Biosynthesis of 18 K and 18 K* POMC in cycloheximide-treated melanotrope cells of black-adapted *Xenopus*

**Figure 4:**
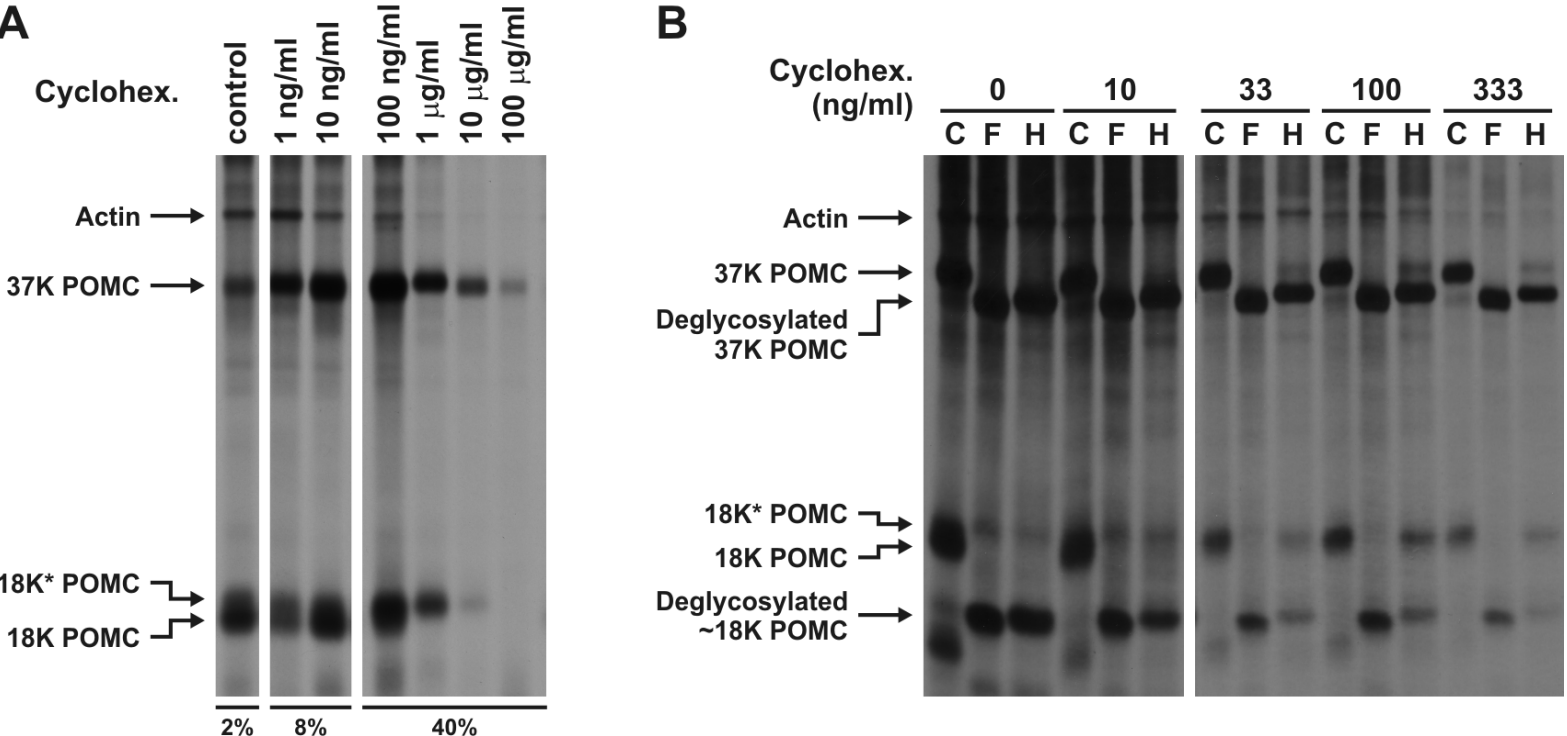
**Glycosylation of POMC in cycloheximide-treated melanotrope cells from black-adapted *Xenopus***. **A**, Neurointermediate lobes (NILs) from black-adapted *Xenopus *were pre-incubated, pulse labelled with [^35^S]methionine/cysteine for 30 minutes and chased for three hours in the absence (control) or presence of cycloheximide (Cyclohex.; drug concentrations indicated above the lanes). Since for the samples of the cycloheximide-treated NILs the intensities of the autoradiographic signals were relatively low, a larger part of the cycloheximide-treated NIL lysates than of the untreated NIL lysates was loaded (indicated below the lanes). The slightly reduced mobility of 37 K POMC in the sample treated with 1 μg/ml cycloheximide was due to a gel problem. **B**, NILs from black-adapted *Xenopus *were pre-incubated, pulsed and chased as in panel A in the absence or presence of the indicated concentration of cycloheximide. Aliquots (10%) of the lysates were control-treated (C) or deglycosylated with Peptidyl N-glycosidase F (PNGaseF; F) or Endoglycosidase H (EndoH; H), and analysed by SDS-PAGE and autoradiography. To compensate for the reduced labelling in the presence of cycloheximide, the right panel was exposed twice as long as the left panel.

The results described above showed that in the Golgi apparatus of BA melanotrope cells only a small portion of 37 K POMC was fully complex glycosylated and sulphated, whereas in the Golgi of WA cells the majority of the POMC molecules was complex glycosylated and sulphated. We hypothesised that the Golgi apparatus of the hyperactive BA melanotrope cells is not able to fully complex glycosylate and sulphate the tremendous amounts of secretory cargo. To test our hypothesis, we pulse labelled and chased BA NILs for three hours in the presence of various concentrations of the protein synthesis-inhibiting drug cycloheximide. In the absence of cycloheximide, POMC biosynthesis and processing proceeded normally (Figure 4A). With increasing concentrations of the inhibitor, protein biosynthesis in the melanotrope cells decreased until a nearly complete inhibition was reached at 100 μg/ml cycloheximide. Interestingly, at low cycloheximide concentrations (up to 10 ng/ml) 18 K POMC constituted the predominant processing product, whereas at 100 ng/ml 18 K* POMC became the more prevalent form and at 1 μg/ml and higher cycloheximide concentrations 18 K* POMC was even the only form produced (Figure 4A). Next, we deglycosylated the newly synthesised proteins and found that the 18 K* POMC produced in the presence of cycloheximide was indeed EndoH-resistant and thus complex glycosylated. In addition, cycloheximide treatment led to a clear increase in EndoH-resistant 37 K POMC (Figure 4B). These findings support our hypothesis that the Golgi apparatus of the hyperactive melanotrope cells is not able to fully complex glycosylate and sulphate the vast amount of secretory cargo it encounters.

## Discussion

In this study, we set out to examine whether hyperactive neuroendocrine secretory cells are able to tune their secretory pathway such that they can comply with the extremely high demands for proper posttranslational modifications of the vast amounts of secretory cargo. We chose as a model cell system the physiologically inducible *Xenopus *melanotrope cells that produce POMC as their main secretory cargo. We show that the hyperactive BA melanotrope cells produced 18 K POMC as the major and 18 K* POMC as a minor POMC-derived product. In contrast, the basally active WA melanotropes produced almost exclusively 18 K* POMC. The difference between the 18 K and 18 K* POMC products concerned only the N-glycan moiety and not the protein backbone. The 18 K POMC product from WA and BA cells as well as most of the 18 K* POMC from BA cells was EndoH sensitive and thus non-complex glycosylated. Remarkably, a significant portion of the 18 K* POMC from WA cells was EndoH resistant and thus complex glycosylated. These findings show that the maturation of N-glycans on the POMC molecule depended on the biosynthetic activity of the cells, and was heterogeneous and incomplete (i.e., not processed to complex glycans), in particular in BA melanotropes. In addition, the extent of POMC tyrosine sulphation differed greatly in the BA and WA cells. Thus, the BA and WA melanotropes secrete a collection of differentially glycosylated and sulphated POMC-derived products. Interestingly, in BA melanotrope cells the rate of biosynthesis of 18 K* POMC was higher than that of 18 K POMC. In addition, 18 K* POMC was released into the incubation medium faster than 18 K POMC, presumably as a result of the faster biosynthesis of 18 K* POMC. The 18 K* POMC product from BA cells may well originate from a pool of 37 K POMC molecules travelling relatively fast through the secretory pathway (restricting the opportunity for post-translational modifications) or, alternatively, bypassing certain machinery-containing subcompartments of the secretory pathway. Clearly, the secretory pathways in the WA and BA melanotrope cells differently fulfil the needs for posttranslational prohormone processing.

The reduced degree of N-glycan processing and sulphation of POMC in BA melanotropes led us to hypothesise that in hyperactive melanotropes the Golgi apparatus is not equipped with sufficient machinery to fully complex glycosylate and sulphate cargo molecules. If true, a reduction in the amount of cargo passing through the Golgi apparatus should increase the fraction of complex glycosylated and sulphated POMC. To test this hypothesis, we chose to reduce POMC biosynthesis by cycloheximide, an inhibitor of protein biosynthesis. In the presence of increasing concentrations of cycloheximide, protein biosynthesis was indeed reduced, whereas the relative amount of complex glycosylated POMC had clearly increased. In summary, we show that in neuroendocrine *Xenopus *melanotrope cells that have been physiologically induced to hyperactive secretory cells the Golgi apparatus is not sufficiently equipped to allow full posttranslational processing of the high amounts of secretory cargo produced. As a result, the hyperactive *Xenopus *melanotropes produce a repertoire of POMC-derived products that are heterogeneously glycosylated and sulphated. The observed heterogeneous posttranslational modifications of secretory cargo do not appear to be restricted to the BA *Xenopus *melanotropes. For example, in bovine posterior pituitaries ~50% of the POMC-derived protein β-lipotropin is sulphated [13], only 5–10% of the N-terminal portion of *Xenopus *POMC is processed to γ-MSH peptides [8] and a 16 K POMC product (the N-terminal part of bovine POMC) is only partly O-glycosylated and cleaved [14]. Unfortunately, these studies did not include an analysis of the effect of physiological cell activation on the posttranslational modifications of POMC.

Since previous studies that have examined the effect of the differential glycosylation and sulphation events on the biosynthesis, intracellular processing, biostability and secretion of POMC are contradictory and inconclusive [5,14-21], the implications -if any- of the affected posttranslational modifications remain unclear, including their effect on the bioactivity of the POMC-derived products. Nevertheless, glycosylation and sulphation are known to alter biological properties, such as the stability and bioactivity of secretory cargo proteins. For example, in frogs the glycans on POMC may provide stability against non-specific proteolysis or degradation [19-21] and the bioactivity of the N-terminal portion of bovine and mouse POMC depends on (the extent of) N- and O-glycosylation [14,22]. Also, the bioactivity of a variety of peptide hormones, e.g. gastrin, cholecystokinin, leu-enkephalin, prolactin, thyrotropin, gonadotropins and follicle stimulating hormone, is known to be influenced by posttranslational modifications [23-30]. Furthermore, a number of signalling pathways, such as the Notch signalling pathway and chemokine signalling and immune regulation pathways, depend on the proper posttranslational modifications of the extracellular signalling molecule or the receptor [31-35]. It is therefore conceivable that the differentially glycosylated and sulphated products secreted by the hyperactive and the basally active melanotropes vary in their bioactivities, and thus that the BA and WA cells have different secretory signals.

## Conclusion

We show that the Golgi apparatus of the hyperactive melanotrope cells is apparently equipped with the appropriate machinery to complex glycosylate and sulphate POMC molecules, but the quantity of the machinery is not sufficient to fully process the vast amounts of POMC passing through the secretory pathway of BA melanotropes. The shortcomings in protein processing events that we have observed in the hyperactive *Xenopus *melanotrope cells may also occur in other hyperactive secretory cell systems, including in neuroendocrine tumour cells and immunoglobulin-secreting plasma cells, suggesting that our findings may well have broader implications.

## Methods

### Animals

Animals were bred and reared in the Central Animal Facility of the Radboud University (Nijmegen, The Netherlands). Animals were background adapted for at least three weeks. All animal experiments were carried out in accordance with the European Communities Council Directive 86/609/EEC for animal welfare and permits RU-DEC 2003–53 and 2007–027 from the animal experiment committee of the Radboud University for the use of *Xenopus laevis*.

### Pulse and pulse-chase analysis

Metabolic cell labelling experiments with [^35^S]methionine/cysteine or with [^3^H]lysine and [^35^S]sulphate and quantification of the newly synthesised products were performed as described previously [10,36]. The *Xenopus *pituitary consists of three parts; the anterior lobe, the intermediate lobe containing the melanotrope cells, and the neuronal lobe consisting of nerve endings originating from the hypothalamus. The anterior lobe can easily be dissected from the pituitary, but the intermediate and neuronal lobes (together the NIL) are intimately associated. Since the neuronal lobe is biosynthetically inactive, we used the NIL for our biosynthetic studies. Dissection of the NIL from WA animals disrupts the inhibitory action of hypothalamic neurons. One of the major factors of hypothalamic origin inhibiting peptide release from the melanotrope cells is dopamine [37]. In order to retain an inhibitory action on peptide secretion during the in vitro incubations of the NILs from WA frogs, we added the dopamine receptor agonist apomorphine to the incubation medium. The removal of all N-linked glycogroups irrespective of their composition by Peptidyl N-glycosidase F was performed as described [10]. For the selective removal of high-mannose glycogroups, protein homogenates were boiled in 85 mM sodium acetate pH 5.5/0.04% SDS/0.08% β-mercaptoethanol/1 mM phenylmethylsulfonyl fluoride (PMSF) for 10 min, cooled to room temperature, 1 μl 5% Triton X-100 and 5 mU Endoglycosidase H (EndoH; Roche) were added, and the samples were incubated overnight at 37°C.

### Statistics

Statistical evaluation was performed using unpaired two-tailed t-tests. In those cases where the variances were significantly different, Welch's correction was used. Differences in mean values were considered of statistical significance if p < 0.05. Calculations were performed using the GraphPad Prism 4 program (GraphPad Software).

## Authors' contributions

JS performed experiments and analysed data, JS and GM designed experiments, JS and GM wrote the paper. Both authors have read and approved the final manuscript.
